# Cannabis and Driving in Older Adults

**DOI:** 10.1001/jamanetworkopen.2023.52233

**Published:** 2024-01-18

**Authors:** Patricia Di Ciano, Tarek K. Rajji, Lauren Hong, Sampson Zhao, Patrick Byrne, Yoassry Elzohairy, Jeffrey R. Brubacher, Michael McGrath, Bruna Brands, Sheng Chen, Wei Wang, Omer S. M. Hasan, Christine M. Wickens, Pamela Kaduri, Bernard Le Foll

**Affiliations:** 1 Institute for Mental Health Policy Research, Centre for Addiction and Mental Health, Toronto, Ontario, Canada; 2 Department of Pharmacology and Toxicology, University of Toronto, Toronto, Ontario, Canada; 3 Dalla Lana School of Public Health, University of Toronto, Toronto, Ontario, Canada; 4 Campbell Family Mental Health Research Institute, Centre for Addiction and Mental Health, Toronto, Ontario, Canada; 5 Department of Emergency Medicine, University of British Columbia, Vancouver, British Columbia, Canada; 6 Ontario Ministry of Transportation, Toronto, Ontario, Canada; 7 Health Canada, Ottawa, Ontario, Canada; 8 Biostatistics Core, Centre for Addiction and Mental Health, Toronto, Ontario, Canada; 9 Institute of Health Policy, Management and Evaluation, University of Toronto, Toronto, Ontario, Canada; 10 Addictions Division, Centre for Addiction and Mental Health, Toronto, Ontario, Canada; 11 Department of Psychiatry, University of Toronto, Toronto, Ontario, Canada; 12 Department of Psychiatry and Mental Health, Muhimbill University of Health and Allied Sciences, Tanzania; 13 Translational Addiction Research Laboratory, Centre for Addiction and Mental Health, Toronto, Ontario, Canada; 14 Institute of Medical Sciences, University of Toronto, Toronto, Ontario, Canada; 15 Acute Care Program, Centre for Addiction and Mental Health, Toronto, Ontario, Canada; 16 Department of Family and Community Medicine, University of Toronto, Toronto, Ontario, Canada; 17 Waypoint Research Institute, Waypoint Centre for Mental Health Care, Penetanguishene, Ontario, Canada; 18 Toronto Dementia Research Alliance, Temerty Faculty of Medicine, University of Toronto, Toronto, Ontario, Canada

## Abstract

**Importance:**

Epidemiological studies have found that cannabis increases the risk of a motor vehicle collision. Cannabis use is increasing in older adults, but laboratory studies of the association between cannabis and driving in people aged older than 65 years are lacking.

**Objective:**

To investigate the association between cannabis, simulated driving, and concurrent blood tetrahydrocannabinol (THC) levels in older adults.

**Design, Setting, and Participants:**

Using an ecologically valid counterbalanced design, in this cohort study, regular cannabis users operated a driving simulator before, 30 minutes after, and 180 minutes after smoking their preferred legal cannabis or after resting. This study was conducted in Toronto, Canada, between March and November 2022 with no follow-up period. Data were analyzed from December 2022 to February 2023.

**Exposures:**

Most participants chose THC-dominant cannabis with a mean (SD) content of 18.74% (6.12%) THC and 1.46% (3.37%) cannabidiol (CBD).

**Main outcomes and measures:**

The primary end point was SD of lateral position (SDLP, or weaving). Secondary outcomes were mean speed (MS), maximum speed, SD of speed, and reaction time. Driving was assessed under both single-task and dual-task (distracted) conditions. Blood THC and metabolites of THC and CBD were also measured at the time of the drives.

**Results:**

A total of 31 participants (21 male [68%]; 29 White [94%], 1 Latin American [3%], and 1 mixed race [3%]; mean [SD] age, 68.7 [3.5] years), completed all study procedures. SDLP was increased and MS was decreased at 30 but not 180 minutes after smoking cannabis compared with the control condition in both the single-task (SDLP effect size [ES], 0.30; *b* = 1.65; 95% CI, 0.37 to 2.93; MS ES, −0.58; *b* = −2.46; 95% CI, −3.56 to −1.36) and dual-task (SDLP ES, 0.27; *b* = 1.75; 95% CI, 0.21 to 3.28; MS ES, −0.47; *b* = −3.15; 95% CI, −5.05 to −1.24) conditions. Blood THC levels were significantly increased at 30 minutes but not 180 minutes. Blood THC was not correlated with SDLP or MS at 30 minutes, and SDLP was not correlated with MS. Subjective ratings remained elevated for 5 hours and participants reported that they were less willing to drive at 3 hours after smoking.

**Conclusions and relevance:**

In this cohort study, the findings suggested that older drivers should exercise caution after smoking cannabis.

## Key Points

**Question:**

What is the association between retail cannabis available to the consumer, driving, and associated blood tetrahydrocannabinol (THC) levels in people over 65 years of age?

**Findings:**

In this cohort study, 31 regular users of cannabis aged 65 to 79 years chose on average high potency (18.74% THC) THC-dominant cannabis. Weaving was increased and speed was decreased at 30 minutes after smoking, which was not correlated with blood THC concentrations; subjective experience and self-reports of impaired driving persisted for 3 hours.

**Meaning:**

These findings suggest that older drivers, even if they regularly use cannabis, show evidence of impaired driving performance after smoking cannabis.

## Teaser

This cohort study evaluates the association between cannabis and simulated driving along with concurrent blood tetrahydrocannabinol levels in older adults in Canada.

## Introduction

Epidemiological studies have established that cannabis increases the risk of a motor vehicle collision.^1,2,3,4,5^ Laboratory studies have demonstrated that this impairment results in increased weaving, slowed reaction time, and compensatory changes in speed and following distance.^6,7,8^ At present, controlled investigations of the effects of cannabis on driving have enrolled younger participants.^6,7,8^ Cannabis use is on the rise in older adults,^9,10,11^ yet the effects of cannabis on driving remain unknown in this population. Older adults may be particularly affected by cannabis, given age-related changes in cognition,^12,13,14,15,16,17,18,19,20^ metabolic changes that may prolong or enhance the effects of cannabis,^21,22,23,24^ and the concomitant use of medications. Conversely, older users of cannabis may have been using cannabis for many years and cannabis may have a diminished impact in this population due to development of tolerance.^25,26,27,28^

Delta-9-tetrahydrocannabinol (THC), the main psychoactive component of cannabis, is believed to be responsible for the impact of cannabis on driving.^29,30,31,32,33^ Based on the success of the deterrence of alcohol-impaired driving, many jurisdictions have adopted per se limits for blood THC levels. These limits set a cutoff of THC in blood that are permitted while driving; these limits vary by jurisdiction but generally range between 0 and 5 ng/mL. Although dose-dependent increases in driving impairment have been observed after cannabis, the exact relationship between blood THC and driving abilities remains debated.^34,35^

The purpose of the present study was to investigate the association between cannabis and driving and blood THC levels in older adults. The present study used an ecologically valid approach in which participants were invited to smoke their usual cannabis in the laboratory. This is important because it is known that legally available cannabis is more potent than the cannabis that has typically been studied in the laboratory.^36^ An ecological approach may yield more valid results into the outcomes of cannabis in everyday situations. In the present study, participants drove the simulator before smoking in a dedicated smoking room and then again at 30 and 180 minutes afterwards; blood was collected for measurement of THC and metabolites as well as cannabidiol (CBD) at the time of the drives. In an ecologically valid control condition, participants relaxed in the dedicated smoking room instead of smoking. We hypothesized that SD of lateral position (SDLP; weaving) after smoking cannabis would be increased compared with the no smoking condition, while reaction time would be slowed and speed decreased. We further hypothesized that measures of driving performance would be associated with blood THC levels.

## Methods

This study was approved by research ethics boards at both the Centre for Addiction and Mental Health and Health Canada. This study was conducted at the Centre for Addiction and Mental Health in Toronto, Canada, between March 2022 and November 2022 with no follow-up period. This study follows the Strengthening the Reporting of Observational Studies in Epidemiology (STROBE) reporting guideline for cohort studies.

### Participants

Adults aged 65 to 79 years were recruited from advertisements placed on public transit and social media. After provision of written informed consent, participants were evaluated for inclusion criteria (see eAppendix 1 in Supplement 1).

### Design and Procedures

This was a within-participants counterbalanced study of the association between cannabis and simulated driving and blood THC. After the telephone interview, potentially eligible participants had an in-person assessment and those eligible participated in a practice session to familiarize them with the study procedures, including an opportunity to drive the simulator. If the participant experienced illness on the simulator, they were given a break. If the illness persisted, they were withdrawn from the study. At this session demographic information was collected, including self-reported race and ethnicity for statistical purposes (participants were provided with the following options: Asian-East [eg, Chinese, Japanese, Korean], Asian-South [eg, Indian, Pakistani, Sri Lankan]. Asian-South East [eg, Malaysian, Filipino, Vietnamese], Black-African [eg, Ghanaian, Kenyan, Somali], Black-Caribbean [eg, Barbadian, Jamaican], Black-North American [eg, Canadian, American] First Nations, Indian-Caribbean [eg, Guyanese with origins in India], Indigenous or Aboriginal not included elsewhere, Inuit, Latin American [eg, Argentinian, Chilean, Salvadoran], Metis, Middle Eastern [eg, Egyptian, Iranian, Lebanese], White-European [eg, English, Italian, Portuguese, Russian], White-North American [eg, Canadian, American], mixed heritage [eg, Black-African and White-North American], other[s], prefer not to answer, do not know). In total, the practice session was about 2 to 3 hours in duration.

This was followed by 2 test sessions of about 7 hours each (5 hours after smoking and 2 hours of baseline), separated by at least 72 hours. Participants were asked to abstain from cannabis, alcohol, and other recreational drugs for 12 hours and received the following 2 conditions in counterbalanced order: (1) cannabis, in which they smoked cannabis in a dedicated negative pressure room; and (2) an ecologically valid control, with no placebo or cannabis, in which they relaxed in the smoking room for approximately 10 minutes.

Before each test session, breathalyzer (Alert J5 model) and saliva sampling (DrugWipe, 5 ng/ml cutoff) were used to confirm self-reported abstinence from alcohol and cannabis in the past 12 hours. In addition, a 14-panel urine screen was used to determine that there was no recent use of other recreational or psychoactive drugs. At the start of each test session, participants were asked about their degree of withdrawal from cannabis, as assessed by the Marijuana Withdrawal Checklist (MWC).^37,38^

Participants then drove the simulator and provided blood for measurement of THC and metabolites before smoking cannabis or relaxing in the smoking room and then again at 30 and 180 minutes after; the timing of blood draws for measurement of THC corresponded to the time of the drives. Cognitive and subjective assessments followed each drive (to be published in a separate report). The visual analog scales (VAS; see eAppendix 2 in Supplement 1 for the definitions) were administered at baseline and then again at 30 minutes, 60 minutes, and hourly until 5 hours after smoking. Before the drive at baseline and at 180 minutes, participants were asked (1) how willing they would be to drive a real vehicle (5-point Likert scale); and (2) how impaired they were at the time to drive^35^ (VAS from 0 to 100). Participants were compensated for their participation in the study. For a schematic of the test day and details of the blood analysis, see eFigure 1 for the test session and eAppendix 3 for analysis of blood in Supplement 1.

### Cannabis

Participants were asked to bring their own legally purchased cannabis to the laboratory. They were asked to smoke the cannabis as a joint with no tobacco. They were given the choice of rolling their own joint or bringing a preroll that was purchased from a retail outlet. Participants were allowed to smoke *ad libitum*. Since this was an ecologically valid design, participants were provided with only a few instructions: they were told to smoke their usual amount to achieve their desired effect and to stop if they felt strange or unwell. They were told that they were not required to smoke the entire cannabis cigarette. The cannabis cigarette was weighed before and after smoking to estimate the amount consumed, with an electronic balance (model VWR-123P) that was calibrated at least weekly in accordance with the manufacturer instructions. The amount of THC and CBD present in the cannabis was determined from the packaging of the cannabis. To estimate the amount of THC smoked, the potency of THC in the cigarette (expressed as a percentage) was multiplied by the change in weight of the cigarette (in mg), and divided by 100.

### Outcomes

The primary outcome was SDLP, the measure most consistently found to be associated with cannabis.^27,33,39,40,41,42,43,44,45^ SDLP (measured in centimeters) is a sensitive measure of the effects of psychoactive drugs on driving and measures the amount of weaving. Secondary outcome measures were mean speed (MS, km/h), brake latency (reaction time; seconds), SD of speed (SDSP), and maximal speed (MAX, km).^30,32,33,46^ MS is the mean speed (km/h) during the drive when asked to maintain a speed of 80 km/h. SDSP represents the variability of speed during a drive. Larger numbers mean that the driver was not able to maintain a consistent speed. MAX is the maximal speed obtained during the drive. Reaction time, or brake latency, is the time for a participant to move their foot from the gas to the brake pedal after a stop sign appears on the road facing them; participants were instructed not to brake when a stop sign appeared that was not facing them. For details of the driving simulator and simulations, see eAppendix 4 in Supplement 1.

All driving outcomes were assessed under both single- and dual-task conditions. In the dual-task condition, participants were required to count backward by threes aloud while driving, starting at a number between 700 and 1000. This condition was included to mimic the situation of driving while distracted or under extra cognitive load and has been shown to be a good measure of driving while distracted.^47,48^

### Statistical Analysis

For details of the sample size calculations, see eAppendix 5 in Supplement 1. Data analyses were performed using statistical software R version 4.0 with packages lme4, car, and lsmeans (R Project for Statistical Computing). To account for the correlation of repeated measures on the participants, mixed-effect models using time (30 minutes and 180 minutes), treatment (no cannabis vs cannabis), their interaction as fixed effects, and individual participants as random effects were adjusted to all outcome measures. The models for the outcome measures also controlled for session order (the sequence of smoking cannabis or no cannabis), baseline blood THC, and the baseline value. The contrasts of the least square means (LSM) of the outcome measures between the treatment groups cannabis at 30 minutes vs no cannabis at 30 minutes, cannabis at 180 minutes vs no cannabis at 180 minutes were provided. Effect sizes (ES, Cohen *d*) are presented for significant effects.

The association between cannabis smoking and blood THC, metabolites of THC, and CBD levels over time were analyzed with mixed-effect models in the cannabis condition. In these models, blood THC or metabolites of THC and CBD levels entered the models as the outcome measures, time (0 minutes, 30 minutes, and 180 minutes) as fixed effect, and individual participants as random effects. Session order was controlled as a covariate. The correlations of SDLP and MS with blood THC in the cannabis group at 30 minutes were tested with correlation analysis (Pearson product-moment correlation).

No adjustment was applied for the comparison of the primary outcome (SDLP) in the cannabis vs the no cannabis groups at 30 or 180 minutes. For secondary driving outcomes, Bonferroni correction was applied. For all other analyses, a significance criterion of *P* < .05 was applied in the 2-sided tests. Data were analyzed from December 2022 to February 2023.

## Results

A participant flow diagram is presented in the Figure. Participant demographics are presented in Table 1. A total of 31 participants (21 male [68%]; 29 White [94%], 1 Latin American [3%], and 1 mixed race [3%]) completed all study procedures. Participants had a mean (SD) age of 68.7 (3.5) years. Participants had been using cannabis a mean (SD) of 40 (16.6) years, and 24 used cannabis more than once a week, primarily for recreational purposes. A total of 25 participants used cannabis recreationally, 1 medically, and 5 both medically and recreationally. No participants were lost to follow-up. There were no missing data except in the no cannabis condition where 2 participants were not able to provide blood at 30 minutes, and 1 was not able to provide blood at 180 minutes. Participants did not report any withdrawal symptoms as determined by the total MWC score (mean [SD], no cannabis session: 1.1 [0.1]; cannabis session: 1.08 [0.11]) and the withdrawal subscore (mean [SD], no cannabis session: 1.1 [0.11]; cannabis session: 1.08 [0.12]). Cannabis was well tolerated and only 1 adverse event (emesis) was reported. For concomitant medications see eTable in Supplement 1.

**Figure.**
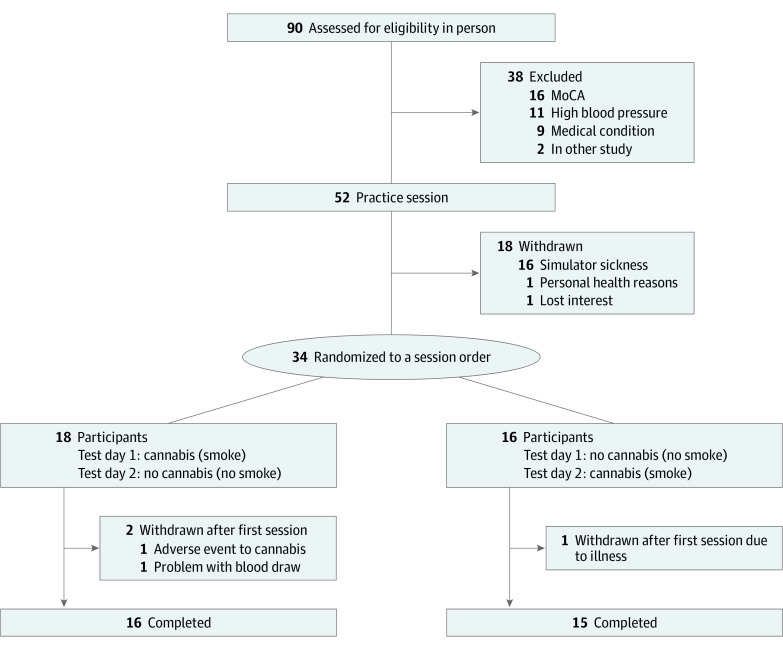
Participant Flow Diagram Illustrating the Various Test Sessions and Visits MoCA indicates Montreal Cognitive Assessment.

**Table 1.** Participant Demographics

Characteristic	Participants, No. (%)
Sex	
Female	10 (32)
Male	21 (68)
Age, mean (SD) [range], y	68.7 (3.5) [65-78]
Race and ethnicity^a^	
Latin American	1 (3)
Mixed race (Black African/White North American)	1 (3)
White European	9 (29)
White North American	20 (65)
Years using cannabis, mean (SD) [range]	40 (16.6) [1-54]
Primary method to administer cannabis	
Joints	15 (48)
Vaporizer	8 (26)
Hand pipe	4 (13)
Edibles	4 (13)
Frequency of cannabis use	
More than once/d	5 (16)
Once/d	9 (29)
5-6 times/wk	6 (19)
3-4 times/wk	2 (6)
Twice/wk	2 (6)
2-3 times/mo	4 (13)
Once/mo	2 (6)
Once every 2 mos	1 (3)
Reason for using cannabis (select one)	
Medical	1 (3)
Recreational	25 (81)
Both	5 (16)
Family income before taxes	
$0-$29 999	4 (13)
$30 000-$59 999	8 (26)
$60 000-$89 999	6 (19)
$90 000-$119 999	4 (13)
$120 000-$149 999	1 (3)
$150 000 or more	5 (16)
Do not know	1 (3)
Prefer not to answer	2 (6)

^a^ Participants were provided with the following options: Asian-East (eg, Chinese, Japanese, Korean), Asian-South (eg, Indian, Pakistani, Sri Lankan). Asian-South East (eg, Malaysian, Filipino, Vietnamese), Black-African (eg, Ghanaian, Kenyan, Somali), Black-Caribbean (eg, Barbadian, Jamaican), Black-North American (eg, Canadian, American) First Nations, Indian-Caribbean (eg, Guyanese with origins in India), Indigenous or Aboriginal not included elsewhere, Inuit, Latin American (eg, Argentinian, Chilean, Salvadoran), Metis, Middle Eastern (eg, Egyptian, Iranian, Lebanese), White-European (eg, English, Italian, Portuguese, Russian), White-North American (eg, Canadian, American), mixed heritage (eg, Black-African and White-North American), other(s), prefer not to answer, do not know.

Characteristics of the cannabis are provided in Table 2. Most participants in the present study chose to smoke THC-dominant cannabis (mean [SD], 18.74% [6.12%]; range, 5.02%-26.87%). Of the 31 participants, 26 smoked cannabis with negligible CBD and more than 14% THC; of these, 17 smoked cannabis with 20% or more of THC. All participants smoked cannabis with THC, and the lowest potency of THC was 5.02%. With respect to CBD, the highest potency of CBD was 12.32% and the lowest potency that contained CBD was 6.90% (mean [SD], 1.46% [3.37%]; range, <1%-12.32%). CBD deemed as negligible by the manufacturer was reported as various ranges on the packaging; for clarity, cannabis with negligible CBD is denoted as less than 1% in Table 2. For calculation of the mean it was given a value of 0.05%, which approximates the amount of CBD. When adjusted for the amount of cannabis smoked, the mean (SD; range) dose of THC inhaled was 56.93 (53.82; 6.11-292.85) mg.

**Table 2.** Characteristics of the Cannabis Smoked in the Laboratory, Presented in Descending Order of Tetrahydrocannabinol (THC) Potency

Participant	THC, %^a^	CBD, %^b^	Change in weight, mg^c^	Time smoking, min	No. of puffs	Amt THC, mg^d^
1	26.87	<1	75	1	4	20.15
2	26.72	<1	1096	20	32	292.85
3	25.23	<1	531	5	8	133.97
4	24.00	<1	237	4	5	56.88
5	23.71	<1	403	10	17	95.55
6	23.70	<1	418	7	19	99.07
7	23.00	<1	274	6	12	63.02
8	22.76	<1	175	3	10	39.83
9	22.40	<1	242	5	7	54.21
10	22.22	<1	383	5	11	85.10
11	22.00	<1	129	3	15	28.38
12	21.98	<1	238	3	9	52.31
13	21.75	<1	261	7	15	56.77
14	21.19	<1	412	6	12	87.30
15	20.40	<1	167	7	19	34.07
16	20.30	<1	192	4	11	38.98
17	20.00	<1	168	5	13	33.60
18	19.28	<1	315	5	9	60.73
19	19.20	<1	504	4	15	96.77
20	18.70	<1	227	8	23	42.45
21	18.70	<1	85	2	4	15.90
22	18.60	<1	254	5	26	47.24
23	18.45	<1	99	2	4	18.27
24	16.82	<1	90	<1	3	15.14
25	15.10	<1	90	2	3	13.59
26	14.50	<1	223	4	8	32.34
27	8.40	6.90	584	9	24	49.06
28	8.40	6.90	863	19	28	72.49
29	6.17	12.32	114	3	7	7.03
30	5.50	8.40	111	3	7	6.11
31	5.02	9.44	312	6	21	15.66
Total, mean (SD) [range]	18.74 (6.12) [5.02-26.87]	1.46 (3.37) [<1-12.32]	299.10 (229.83) [75-1096]	5.61 (4.31) [<1-20]	12.94 (7.87) [3-32]	56.93 (53.82) [6.11-292.85]

Abbreviation: CBD, cannabidiol.

^a^ Potency of the THC in cannabis as indicated on the packaging.

^b^ Potency of the CBD in the cannabis as indicated on the packaging (CBD deemed as negligible by the manufacturer was reported as varying ranges on the packaging; for clarity, cannabis with negligible CBD is denoted as less than 1%).

^c^ Change in weight of the cigarette after smoking.

^d^ The amount of THC consumed, calculated from the potency of THC and change in weight.

### Primary Outcome SDLP

After smoking cannabis, significant differences were observed contrasting the LSM at 30 minutes between the cannabis and the no cannabis condition for SDLP under single-task (ES = 0.30; *b* = 1.65; 95% CI, 0.37-2.93; *t*_90.4_ = 2.53; *P* = .01). The same outcome was observed for SDLP under dual-task conditions (ES = 0.27; *b* = 1.75; 95% CI, 0.21-3.28; *t*_89.6_ = 2.24; *P* = .03) (Table 3).

**Table 3.** Descriptive Results for the Primary and Secondary Driving Outcomes

Outcome	Mean (SD)					
	No cannabis			Cannabis		
	Baseline	30 min	180 min	Baseline	30 min	180 min
Single task						
SDLP, cm	30.5 (5.7)	30.2 (5.5)	30.4 (5.3)	31.1 (5.5)	32.3 (4.6)^a^	31.7(5.3)
MS, km/h	82.4 (3.3)	83.3 (4.3)	83.3 (4.7)	81.9 (3.6)	80.4 (3.9)^a^	82.3 (3.9)
SDSP, km/h	5.3 (1.9)	5.1 (1.9)	4.9 (2.0)	5.1 (1.7)	5.6 (2.3)	5.2 (1.4)
MAX, km/h	95.0 (5.2)	95.6 (6.3)	96.3 (7.7)	94.3 (6.5)	92.8 (6.1)	95.5 (5.8)
RT, sec	.94 (.14)	.94 (.12)	.94 (.12)	.94 (.12)	.94 (.12)	.94 (.13)
Dual task						
SDLP, cm	29.7 (5.0)	30.4 (6.5)	30.0 (5.3)	29.9 (5.2)	32.3 (5.1)^a^	31.4 (6.4)
MS, km/h	86.0 (6.3)	85.9 (6.7)	84.8 (5.7)	85.7 (6.5)	82.5 (6.7)^a^	86.4 (7.3)
SDSP, km/h	6.3 (2.9)	5.8 (2.2)	6.4 (2.9)	6.9 (3.7)	6.5 (2.2)	6.7 (2.6)
MAX, km/h	100.7 (9.1)	100.0 (9.5)	100.0 (8.0)	102.5 (10.5)	98.9 (8.8)	101.3 (9.2)
Blood, ng/mL						
THC	2.55 (4.3)	2.38 (4.0)	2.45 (3.9)	2.38 (3.6)	25.57 (28.2)^b^	4.75 (6.3)
THC-COOH	29.29 (41.5)	27.30 (38.0)	24.98 (32.0)	31.54 (48.3)	52.21 (64.4)^b^	36.88 (46.2)
THC-11-OH	1.06 (1.3)	1.02 (1.3)	1.04 (1.2)	1.09 (1.5)	5.41 (6.8)^b^	2.17 (3.1)
CBD	0.33 (0.5)	0.30 (0.5)	0.23 (0.4)	0.31 (0.6)	3.24 (7.9)^b^	0.59 (0.9)

Abbreviations: CBD, cannabidiol; COOH, delta-9-carboxy-tetrahydrocannabinol; MAX, maximum speed; MS, mean speed; RT, reaction time; SDLP, standard deviation of lateral position; SDSP, standard deviation of speed; THC, tetrahydrocannabinol; THC-11-OH, 11-hydroxy-THC.

^a^ *P* < .05, least square means contrast to the no cannabis condition at that time point.

^b^ *P* < .05, least square mean contrasts from baseline.

### Secondary Outcomes

#### Driving

After smoking cannabis, significant differences after multiple-comparison adjustment were observed for MS contrasting the LSM at 30 minutes between the cannabis and the no cannabis conditions under single-task (ES = −0.58; *b* = −2.46; 95% CI, −3.56 to −1.36; *t*_89.9_ = −4.37; *P* < .001) and dual-task conditions (ES = −0.47; *b* = −3.15; 95% CI, −5.05 to −1.24; *t*_89.6_ = −3.24; *P* = .01), with MS being lower in the cannabis compared with the no cannabis condition. Comparisons for reaction time, SDSP (single- or dual-task) or MAX (single- or dual-task) were not significant (Table 3).

#### Blood THC and Metabolites and CBD

For all measures, levels of THC, metabolites, and CBD were significantly higher at 30 minutes than baseline in the cannabis condition (THC: *t*_60_ = 6.51; *P* < .001; delta-9-carboxy-THC: *t*_60_ = 4.47; *P* < .001; 11-hydroxy-THC: *t*_60_ = 5.69; *P* < .001; CBD: *t*_60_ = 2.65; *P* = .01) but not in the no cannabis condition (Table 3).

#### Association of Driving With Blood THC and CBD

Correlation analysis between THC values at 30 minutes and driving measures at 30 minutes did not reveal any significant correlation under either the single- or dual-task conditions for SDLP (*r* = 0.147; *t*_29_ = 0.802; *P* = .43; SDLP dual-task: *r* = 0.027; *t*_29_ = 0.145; *P* = .89) or MS (*r* = 0.206; *t*_29_ = 1.135; *P* = .27; MS dual-task: *r* = 0.056; *t*_29_ = 0.305; *P* = .76). For the association of SDLP to MS, see eAppendix 6 in Supplement 1. Cannabis increased ratings of subjective experience and perceived impairment to drive (see eAppendix 2, eFigure 2, and eAppendix 7 in Supplement 1).

## Discussion

In the present study, it was found that SDLP (weaving) was increased and MS was decreased at 30 minutes but not 180 minutes after smoking cannabis. Blood THC was increased 30 minutes after smoking, but THC levels were not correlated with SDLP or MS. The mean potency of cannabis chosen by participants (18.74% THC) represents a higher potency than previously studied.

In the present study, smoked cannabis increased SDLP and decreased mean speed 30 minutes after smoking under both single- and dual-task conditions. Change in SDLP is the measure most consistently found to be associated with cannabis, and our results are therefore consistent with past observations.^27,33,39,40,41,42,43,44,45^ The mean difference in SDLP of about 2.0 to 2.5 cm is similar to that observed in on-road studies after intoxicating doses of alcohol (breath alcohol concentration [BrAC] of 0.05%),^49,50^ and thus represents a small yet statistically significant and clinically relevant increase. For the dual task condition, although significant, it should be noted that the changes were less than that observed under a BrAC of 0.05%. The decrease in MS is also consistent with our past findings^49,51^ and with the observations of others.^7,30,33,46^ It has been suggested that decreased speed after cannabis is a compensatory change in driving^52^ in response to a participant’s awareness that they are intoxicated. Collectively, the data suggest that cannabis has some impact on driving in older adults.

Although the increase in SDLP was statistically significant, it is smaller than the changes we have observed in the past.^49^ In this regard, it should be mentioned that, although most participants smoked high potency cannabis (18.74%), they titrated to a mean of 56.93 mg of THC. This is less than we have observed in past studies^51,53^ and the relatively small change in SDLP may reflect the lower THC concentrations smoked. In addition, as most participants in the present study had been using cannabis regularly for many decades, it is possible that many participants had partial tolerance to the effects of THC. In any event, our findings suggest that cannabis affects driving in older adults, and even frequent long-term older users should exercise caution and not drive after use of cannabis.

Blood THC was increased after smoking cannabis; metabolites of THC were also increased. There was no correlation between blood THC concentration and SDLP or MS. This finding differs from some of the published literature that suggests that there is a dose-response association between cannabis and driving.^29,30,32,33^ However, the lack of correlation between driving and blood THC fits within emerging evidence that there is not a linear relationship between the 2.^34,35^ It may be possible that, for the smoked route, driving is impacted when THC exceeds the legal threshold^34^; however, in the present study, THC levels were above the legal threshold for most participants, and thus analysis of the association between legal thresholds and performance was precluded. The blood THC levels analyzed in this study corresponded to the time of the drive at 30 minutes after smoking and it should also be considered that blood THC levels at different times after smoking may be related to driving. Future studies with a full pharmacokinetic curve may help to unravel the nuances of the relationship between blood THC and driving.

In this study, the associations between cannabis and driving were apparent at 30 minutes but not 180 minutes after smoking. Despite this, participants still rated their ability to drive at 180 minutes as impaired. It is possible that beliefs about driving ability may reflect the time course of subjective experience, which can persist for hours, as evidenced in our VAS.^49,54,55^ Alternatively, it is possible that participants were impaired at 180 minutes but the simulator lacks the sensitivity to detect small changes. Given that this study was not blinded, it may also be possible that the participant ratings were influenced by a desire to please the experimenter.

### Limitations

One limitation of this study is the limited generalizability of the findings given that the participants were mostly White and more than half were male. To conduct rigorous analyses of effects of sex or ethnicity, large sample sizes are needed, and this is therefore a consideration for future studies. One aspect of this study that nevertheless increases its generalizability is the fact that a number of comorbidities and concomitant medications were noted, making this study applicable to a broad range of conditions. One further limitation of the present study may be limited sensitivity of a driving simulator to detect nuanced changes in driving many hours after smoking. In the present study, driving was not impaired at 180 minutes but participants rated their driving ability as diminished. Future studies will need to investigate driving and impairment with a number of different sensitive proxies.

## Conclusions

The present study provides an ecologically valid demonstration that cannabis can impair driving in older adults when they smoke their usual product. Consistent with emerging data, blood THC level was not correlated with driving behavior. Older drivers should refrain from using cannabis when contemplating operation of a motor vehicle.

## Notes

Supplement 1. **eAppendix 1.** Inclusion and Exclusion Criteria **eFigure 1.** Schematic of the Test Sessions **eAppendix 2.** Visual Analog Scales **eAppendix 3.** Analysis of Blood **eAppendix 4.** Driving Simulator and Driving Simulations **eAppendix 5.** Sample Size Calculations **eTable.** List of Concomitant Medications in the Study **eAppendix 6.** Association of SDLP With MS **eFigure 2.** Descriptive Means (SD) on Measures of the Visual Analog Scale **eAppendix 7.** Perceived Willingness to Drive

Supplement 2.
Data Sharing Statement
