# Supplementary material for: Cannabis and Driving in Older Adults
Source: JAMA Netw Open. 2024 Jan 18;7(1):e2352233. doi: 10.1001/jamanetworkopen.2023.52233 (PMC10797455; doi:10.1001/jamanetworkopen.2023.52233)
Supplement: Supplement 1. — eAppendix 1. Inclusion and Exclusion Criteria eFigure 1. Schematic of the Test Sessions eAppendix 2. Visual Analog Scales eAppendix 3. Analysis of Blood eAppendix 4. Driving Simulator and Driving Simulations eAppendix 5. Sample Size Calculations eTable. List of Concomitant Medications in the Study eAppendix 6. Association of SDLP With MS eFigure 2. Descriptive Means (SD) on Measures of the Visual Analog Scale eAppendix 7. Perceived Willingness to Drive [file jamanetwopen-e2352233-s001.pdf]

Supplemental Online Content

Di Ciano P, Rajji TK, Hong L, et a. Cannabis and driving in older adults. *JAMA Netw Open*. 2024;7(1):e2352233. doi:10.1001/jamanetworkopen.2023.52233

eAppendix 1. Inclusion and Exclusion Criteria

eFigure 1. Schematic of the Test Sessions

eAppendix 2. Visual Analog Scales

eAppendix 3. Analysis of Blood

eAppendix 4. Driving Simulator and Driving Simulations

eAppendix 5. Sample Size Calculations

eTable. List of Concomitant Medications in the Study

eAppendix 6. Association of SDLP With MS

Figure 2. Descriptive Means (SD) on Measures of the Visual Analog Scale

eAppendix 7. Perceived Willingness to Drive

eReferences

This supplemental material has been provided by the authors to give readers additional information about their work.

© 2024 Di Ciano et al. *JAMA Network Open.*

eAppendix 1. Inclusion and Exclusion Criteria

Inclusion criteria: 1) use of cannabis recreationally or medically at least once a month, with use of smoked cannabis at least once in the previous 6 months; 2) 65 to 79 years old; 3) holds a valid regular class driver’s licence; 4) willing to abstain from using cannabis, alcohol and other recreational drugs for 12 hours prior to the test session; 5) willing to bring legally purchased cannabis to the lab to smoke during the session and to prepare it themselves (or bring a pre-roll); 6) normal heart rate and blood pressure as evaluated by the study physician; 7) provides written and informed consent

Exclusion criteria: 1) unstable medical or psychiatric conditions as determined by the study team (Structured Clinical Interview for DSM (SCID no current unstable diagnosis); Montreal Cognitive Assessment (MoCA score of 26 or above); Cumulative Illness Rating Scale Geriatrics (low score on CIRS-G); 2) taking medications or have any medical condition that may affect driving or for which cannabis is contraindicated, including opioids for pain (self-report and urine toxicology); 3) self-report of cognitive decline; 4) self-report of cognitive diagnosis; 5) participation in other research studies

© 2024 Di Ciano et al. *JAMA Network Open.*

eFigure 1. Schematic of the Test Sessions

VAS: Visual Analog Scale; Questions: Driving willingness and impaired driving questions

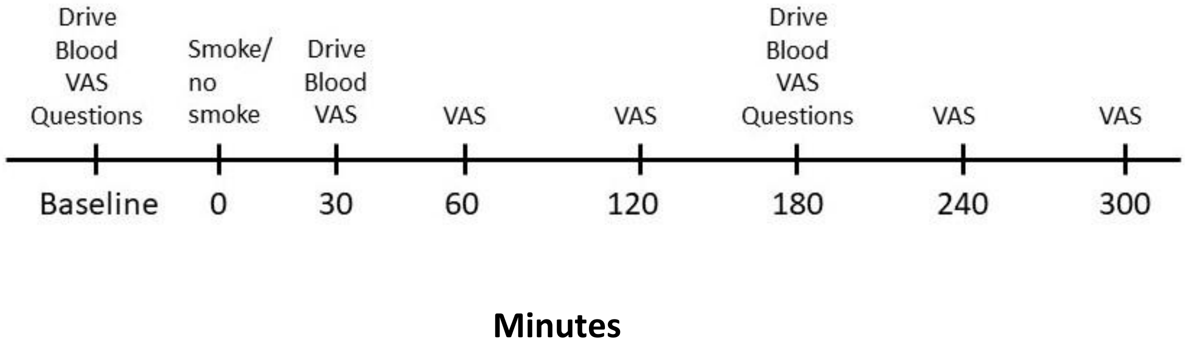

© 2024 Di Ciano et al. *JAMA Network Open.*

eAppendix 2. Visual Analog Scales

Visual analog scales included were: I feel this effect (EFFECT); I feel the good effects (GOOD); I feel the bad effects (BAD); I feel drowsy (DROW); I feel nauseated (NAUS). For the VAS, due to the number of comparisons, efforts were made to limit familywise error. Thus, to be consistent with other outcomes, only the difference in least square means between conditions at 30 minutes and 180 minutes after smoking were analyzed. For all measures, there were differences between conditions at 30 minutes (EFFECT: $t(91.5)=21.43$, $p=0$; GOOD: $t(89.4)=22.14$, $p=0$; BAD: $t(89.7)=5.61$, $p<0.001$; DROW: $t(90.8)=5.45$, $p<0.001$; NAUS: $t(89.9)=2.21$, $p=0.03$). All but NAUS was different at 180 minutes (EFFECT: $t(91.5)=14.36$, $p=0$; GOOD: $t(89.4)=18.49$, $p=0$; BAD(89.7): $t=2.09$, $p<0.04$; DROW: $t(90.8)=3.78$, $p=0.001$).

© 2024 Di Ciano et al. *JAMA Network Open.*

eAppendix 3. Analysis of Blood

Each sample of blood (10mL) was collected in a lavender top test tube and transferred to cryotubes to be stored in the freezer (-80 degrees Celsius) until shipment to the blood processing facility (Dynacare). Extraction and analysis of THC, COOH-THC, OH-THC and CBD in whole blood was performed according to a method developed in-house by Dynacare. Briefly, 100 µL of each sample was mixed with methanol containing the Cannabinoids Working Internal standard (IS), allowing for precipitation. Samples were vortexed for 60 seconds, then allowed to equilibrate at room temperature for 10 minutes. Subsequently, samples were centrifuged at 4500 RPM for 5 minutes. The supernatant was transferred into an HPLC vial and injected onto the Prominence HPLC System (Shimadzu) followed by subsequent analysis on the 6500+ QTRAP LC-MS/MS (SCIEX). All analytical data were collected and processed using Analyst 1.6.2. The concentration of cannabinoids in the samples were determined using linear regression with a weighting factor of 1/x. The limit of quantitation (LoQ) for all cannabinoids was 0.2 ng/mL, with an analytical measuring range of 0.2 to 500 ng/mL. For all samples with values of <0.2 ng/mL a value of 0.1 ng/mL was substituted for analysis.

© 2024 Di Ciano et al. *JAMA Network Open.*

eAppendix 4. Driving Simulator and Driving Simulations

Driving Simulator

Customized driving scenarios were programmed using the Virage VS500M simulator to collect data on the outcome measures. The VS500M simulator features the driver's side instrument cluster, steering wheel, controls, and centre console of a General Motors compact car. The brake and accelerator pedals, along with the steering wheel, provide dynamic force feedback. The visual system consists of three 55-inch screens providing a 180° field of view in the front, and two 17-inch side displays providing visual feedback for the left and right blind zones [1].

© 2024 Di Ciano et al. *JAMA Network Open.*

Driving Simulations

For the primary outcome, eight custom scenarios were all programmed on the same 9 km 2-lane rural highway. Participants were instructed to maintain a speed of 80 km/h and drive in the center of the lane to the best of their ability, to stay on the main road and drive as they normally would and to interact with other vehicles and obstacles as they would in the real world. Data was recorded at a frequency of 10 hertz. The same scenario was also used to collect data on other outcome measures such as mean speed (MS), standard deviation of speed (SDSP) and maximal speed (MAX). This scenario did not contain any obstacles, or hazards on the road that required any maneuvering from the driver. Also, data from the first and the last 100 meters of the scenario was filtered out from the final outcome calculations to maintain a clean dataset.

Six separate driving scenarios were programmed to measure reaction time in terms of brake pedal latency. These scenarios consisted of an endless 4-lane highway where participants were instructed to drive at 100km/h, while remaining in the second lane to the right. When presented with a true stop sign (stop sign facing them) they were to come to a complete stop as quickly as possible. When presented with a false stop sign (stop sign facing away from them) they were to maintain their speed. During each trial a total of 10 stop signs appeared suddenly at the far-right lane at random intervals, 7 of them were true and 3 of them were false. Order of the true and false stop signs was different between the twelve scenarios. Also, the stop signs were programmed to only appear if the driver had their foot on the gas pedal (greater than 0.2 depression on a scale of 0-1, 20% depression) for consistency purposes. Data was recorded at a frequency of 60 hertz for this scenario.

© 2024 Di Ciano et al. *JAMA Network Open.*

eAppendix 5. Sample Size Calculations

The target sample size was based on our previous study of simulated driving performance under the influence of cannabis [1]. Using SDLP to compute the sample size, to achieve 80% power considering an alpha of 0.05 and a Cohen's f of 0.28 (medium effect), we needed to complete 31 participants. For a more intuitive paired comparison, the minimum detectable effect size is 0.52 (Cohen's d) for this sample size. Given that the standard deviation of the placebo condition at 30 minutes is 5.5 (single task), the minimum detectable difference in SDLP is about 2.86, which is comparable to the international standard of 2.4. This sample size is in line with that of previous studies that have demonstrated effects of cannabis on simulated driving [2-4].

© 2024 Di Ciano et al. *JAMA Network Open.*

eTable 1. List of Concomitant Medications in the Study

The purpose of use is as indicated by the participant.

Taking no medications:		11
Taking one medication:		1
Taking two medications:		4
Taking three medications:		4
Taking more than three medications:		11
Acid reflux	Pantoprazole	5
	Rabeprazole	2
Anti-inflammatory	Meloxicam	1
	Celecoxib	1
Anxiety	Venlafaxine	1
	Trintellix	1
Asthma	Asthma spray	2
	Flovent	1
	Symbicort	1
Atrial Fibrillation	Metoprolol	1
Benign Prostatic Hyperplasia	Dutasteride	1
	Tamsulosin	2
Blood thinner	Aspirin	2
	Eliquis	2
Depression	Cipralex	2
	Citalopram	1
Diabetes	Jardiance	1
	Metformin	4
	Tradjenta (Linagliptin)	1
Dry eye	Restasis	1
Erectile Dysfunction	Sildenafil	2
Estrogen Suppressor	Letrozole	1
Fibromyalgia	Pregabalin	1
Gastroesophageal Reflux Disease	Esomeprazole	1
Gout	Allopurinol	1
Headaches	Tylenol	1
High Cholesterol	Atorvastatin	3
	Ezetimibe	2
	Ezetrol	1
	Rosuvastatin	5
	Simvastatin	1
High Heart Rate	Bisoprolol	1
Hives	Antihistamine	1

© 2024 Di Ciano et al. *JAMA Network Open.*

Hormonal regulation	Hormonal vaginal insert	1
Hypertension	Chlorthalidone	1
	Enalapril	1
	Ramipril	1
	Amlodipine	3
	Candesartan/HCT	1
	Candesartan	1
	Hydrochlorothiazide	1
	Irbesartan	2
	Perindopril	3
Hypothyroidism	Levothyroxine sodium	4
Overactive Bladder	Fesoterodine	1
	Mirabegron	1
Pain	Naproxen	1
Sleep Apnea	Zopiclone	2
Stiff Muscles	Robaxacet	1
Stomach Acid, Barrett's Esophagus	Omeprazole	1
Ulceritive Colitis	Entyvio (Vedolizumab)	1
Unknown	Rosuvastatin	1
Nasal		1
Topical		2

© 2024 Di Ciano et al. *JAMA Network Open.*

eAppendix 6. Association of SDLP With MS

Due to the change in SDLP and MS observed, it was interest to determine whether these variables were related. Correlation analysis between SDLP and MS did not reveal any significant correlations at baseline (r=0.05, p=.78), 30 minutes (r=-0.065, p=0.73) or 180 minutes (r=0.176, p=0.34).

© 2024 Di Ciano et al. *JAMA Network Open.*

eFigure 2. Descriptive Means (SD) on Measures of the Visual Analog Scale

Values are presented for baseline and throughout the session after smoking cannabis (solid lines) or after relaxing in the smoking room in the control condition (stippled lines). ***p<0.05 all contrasts to the no cannabis condition at that time point; +p<0.05 contrasts to the no cannabis condition in BAD and DROW; to limit familywise error only the 30 minute and 180 minute time points were analyzed. EFFECT: I feel this effect; GOOD: I feel the good effects; BAD: I feel the bad effects; DROW: I feel drowsy; NAUS: I feel nauseated.

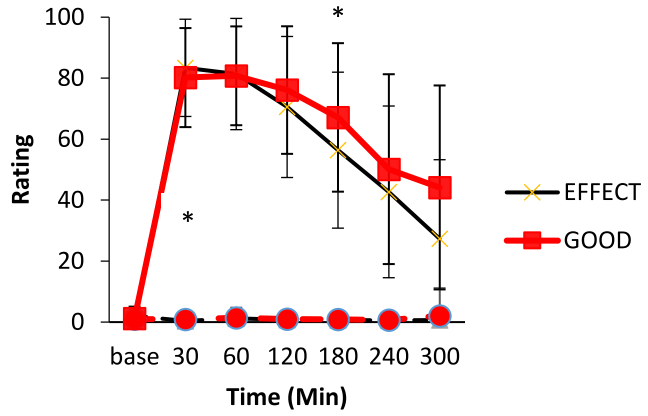

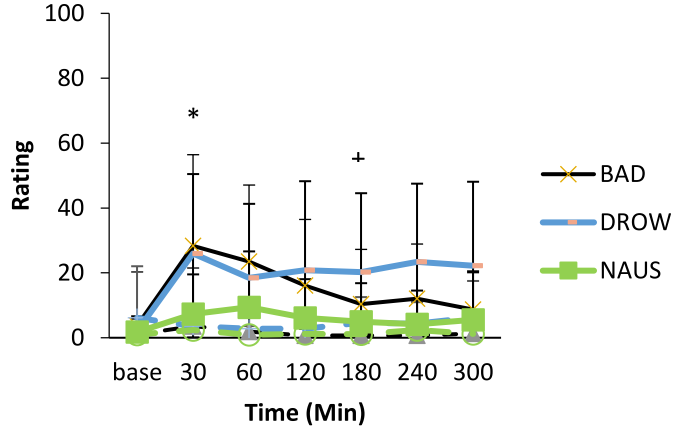

© 2024 Di Ciano et al. *JAMA Network Open.*

eAppendix 7. Perceived Willingness to Drive

For self-rated driving willingness and impairment, comparisons were made between the Cannabis and No Cannabis conditions with a Wilcoxon non-parametric test for paired samples, at each time point (baseline and 180 minutes after smoking). When asked how willing the participants are to drive, there was no difference between conditions at baseline, but a significant difference was found at the 180-min mark such that participants were less willing to drive after smoking cannabis ($Z=-4.155$, $p<0.001$). When asked how impaired they are, participants rated their level of impairment to be greater 180 minutes after smoking cannabis ($Z=-4.705$, $p<0.001$).

© 2024 Di Ciano et al. *JAMA Network Open.*

eReferences

1. Brands B, Mann RE, Wickens CM, et al. Acute and residual effects of smoked cannabis: Impact on driving speed and lateral control, heart rate, and self-reported drug effects. *Drug Alcohol Depend.* 2019;205:107641.
2. Doroudgar S, Mae Chuang H, Bohnert K, Canedo J, Burrowes S, Perry PJ. Effects of chronic marijuana use on driving performance. *Traffic Inj Prev.* 2018;19(7):680-686.
3. Arkell TR, Lintzeris N, Kevin RC, et al. Cannabidiol (CBD) content in vaporized cannabis does not prevent tetrahydrocannabinol (THC)-induced impairment of driving and cognition. *Psychopharmacology (Berl).* 2019.
4. Hartley S, Simon N, Larabi A, et al. Effect of Smoked Cannabis on Vigilance and Accident Risk Using Simulated Driving in Occasional and Chronic Users and the Pharmacokinetic-Pharmacodynamic Relationship. *Clin Chem.* 2019.

© 2024 Di Ciano et al. *JAMA Network Open.*
