# Supplementary material for: Cannabis and Driving in Older Adults
Source: JAMA Netw Open. 2024 Jan 18;7(1):e2352233. doi: 10.1001/jamanetworkopen.2023.52233 (PMC10797455; doi:10.1001/jamanetworkopen.2023.52233)
Supplement: Supplement 2. — Data Sharing Statement [file jamanetwopen-e2352233-s002.pdf]

Data Sharing Statement

Di Ciano. Cannabis and Driving in Older Adults. *JAMA Netw Open*. Published January 18, 2024. doi:10.1001/jamanetworkopen.2023.52233

Data
Data available: Yes
Data types: Deidentified participant data
How to access data: Please send request to Patricia Di Ciano: patricia.diciano@camh.ca
When available: With publication

Supporting Documents
Document types: Informed consent form; protocol
How to access documents: With request to patricia.diciano@camh.ca
When available: With publication

Additional Information
Who can access the data: Researchers whose proposed use of the data has been approvded
Types of analyses: For research purposes
Mechanisms of data availability: After approval of a proposal
